# Functional maintenance of calcium store by ShcB adaptor protein in cerebellar Purkinje cells

**DOI:** 10.1038/s41598-020-71414-y

**Published:** 2020-09-02

**Authors:** Sho Kakizawa, Yasushi Kishimoto, Shinichiro Yamamoto, Kazuko Onga, Kunihiko Yasuda, Yoshiaki Miyamoto, Masahiko Watanabe, Ryuichi Sakai, Nozomu Mori

**Affiliations:** 1 Department of Anatomy and Neurobiology, Graduate School of Biomedical Sciences, Nagasaki University, Nagasaki, 852-8523 Japan; 2 Department of Biological Chemistry, Graduate School of Pharmaceutical Science, Kyoto University, Sakyo-ku, Kyoto, 606-8501 Japan; 3 Department of Biophysics, Kagawa School of Pharmaceutical Sciences, Tokushima Bunri University, Sanuki, Kagawa 769-2193 Japan; 4 Department of Pharmaceutical Therapy and Neuropharmacology, Graduate School of Medicine and Pharmaceutical Sciences, University of Toyama, Toyama, 930-0194 Japan; 5 Department of Anatomy, Hokkaido University School of Medicine, Sapporo, 060-8638 Japan; 6 Department of Biochemistry, Kitasato University School of Medicine, Sagamihara, Kanagawa 252-0373 Japan; 7 Faculty of Medicine, Fukuoka International University of Health and Welfare, Fukuoka, 814-0001 Japan; 8 Present Address: Division of Pharmacology, Faculty of Pharmaceutical Sciences, Teikyo Heisei University, Nakano-ku, Tokyo, 164-8530 Japan; 9 Present Address: Department of Occupational Therapy, Faculty of Fukuoka Medical Technology, Teikyo University, Omuta, 836-8505 Japan

**Keywords:** Neuroscience, Physiology

## Abstract

Intracellular Ca^2+^ levels are changed by influx from extracellular medium and release from intracellular stores. In the central nervous systems, Ca^2+^ release is involved in various physiological events, such as neuronal excitability and transmitter release. Although stable Ca^2+^ release in response to stimulus is critical for proper functions of the nervous systems, regulatory mechanisms relating to Ca^2+^ release are not fully understood in central neurons. Here, we demonstrate that ShcB, an adaptor protein expressed in central neurons, has an essential role in functional maintenance of Ca^2+^ store in cerebellar Purkinje cells (PCs). ShcB-knockout (KO) mice showed defects in cerebellar-dependent motor function and long-term depression (LTD) at cerebellar synapse. The reduced LTD was accompanied with an impairment of intracellular Ca^2+^ release. Although the expression of Ca^2+^ release channels and morphology of Ca^2+^ store looked intact, content of intracellular Ca^2+^ store and activity of sarco/endoplasmic reticular Ca^2+^-ATPase (SERCA) were largely decreased in the ShcB-deficient cerebellum. Furthermore, when ShcB was ectopically expressed in the ShcB-KO PCs, the Ca^2+^ release and its SERCA-dependent component were restored. These data indicate that ShcB plays a key role in the functional maintenance of ER Ca^2+^ store in central neurons through regulation of SERCA activity.

## Introduction

Ca^2+^ signal is one of the most important intracellular signals. Intracellular Ca^2+^ levels are affected by influx from extracellular fluid and release from intracellular stores such as the endoplasmic reticulum (ER). Along with Ca^2+^ influx, Ca^2+^ release is essential for a wide range of cellular responses, including muscle contraction, hormone secretion, and immune responses^1–4^. Therefore, functional maintenance of ER Ca^2+^ stores is essential for most eukaryotic cells^5–7^. In the central nervous systems, Ca^2+^ release is involved in various physiological events, such as excitation and transmitter release^6,8^. Therefore, stable Ca^2+^ release in response to stimuli is critical for proper functioning of the nervous system. However, the regulatory mechanisms behind Ca^2+^ release have not been fully understood in neurons in the central nervous system (CNS). Specifically, signaling molecules involved in functional maintenance of Ca^2+^ stores in CNS neurons are yet to be ascertained.

Of the various candidates thought to contribute to ER Ca^2+^-store maintenance, we aim to explore the involvement of signaling adaptor proteins^9^ because previous studies have reported the possible binding of adaptor proteins to ER proteins that regulate intracellular Ca^2+^ release systems in non-neuronal cells. For example, in skeletal and cardiac muscle cells, insulin receptor substrate (IRS)-1 and IRS-2 have been shown to bind to sarco/endoplasmic reticulum Ca^2+^-ATPase (SERCA)-1 and SERCA-2, respectively^10^. Another adaptor protein, α-fodrin, also binds SERCA-2 in astrocytes^11^. The signaling adaptor Shc emerged at the dawn of metazoan evolution^12^. In mammals, at least for mouse and human genomes, four Shc loci *Shc1*, *Shc2*, *Shc3* and *Shc4* are present and encode the proteins ShcA, ShcB (also called Sck, Sli), ShcC (also called N-Shc, Rai) and ShcD, respectively^13,14^. ShcA is ubiquitously expressed throughout most tissues, but not in the mature brain. In the brain, ShcA expression gradually diminishes during development^15,16^, and ShcB and ShcC expression becomes dominant in mature neurons^17^. ShcB expression is more strongly confined to the nervous system, and ShcC appears to be exclusively expressed in neuronal cells^16,18–21^. In human and rat CNS, the expression profiles of ShcB and ShcC mRNAs overlap considerably, although there are some reports of distinct localization; in the adult rat brain, the level of ShcC mRNA is highest in the thalamus, whereas that of ShcB is highest in the hippocampus^19^. ShcD also shows relatively widespread distribution in the brain and spinal cord of adult rats, with prominent levels of expression throughout the olfactory bulb, as well as in sub-structures of the cerebellum and hippocampus, including the subgranular zone^22^. A genetic deletion of ShcA results in embryonic lethality due to malformation of the cardiovascular system^23^, whereas genetic deletion mutants for ShcB and/or ShcC, which have high expression in the nervous system, are viable^16,17^. ShcC null mice appear not to show gross anatomical abnormalities, but ShcB-deficient mice exhibit a loss of peptidergic and nonpeptidergic sensory neurons. In addition, mice lacking both ShcB and ShcC exhibit a significant additional loss of neurons within the superior cervical ganglia. Therefore, it is assumed that ShcB and ShcC take over the roles of phosphotyrosine (pTyr)-adaptor, at least partially, in mature neurons^14,16,19^. However, the functional significance of ShcB or ShcC is not fully understood, even though they retain similar pTyr-binding domains, i.e., phosphotyrosine-binding (PTB) and Src-homology 2 (SH2) domains for signal outputs, along with collagen-homology (CH)1 and CH2 domains^24–26^.

Here, we show that ShcB is essential for the functional maintenance of Ca^2+^ store in CNS neurons through the regulation of SERCA activity, particularly in cerebellar Purkinje cells (PCs) in mice.

## Results

### Cerebellar dysfunction in ShcB-deficient mice

**Figure 1:**
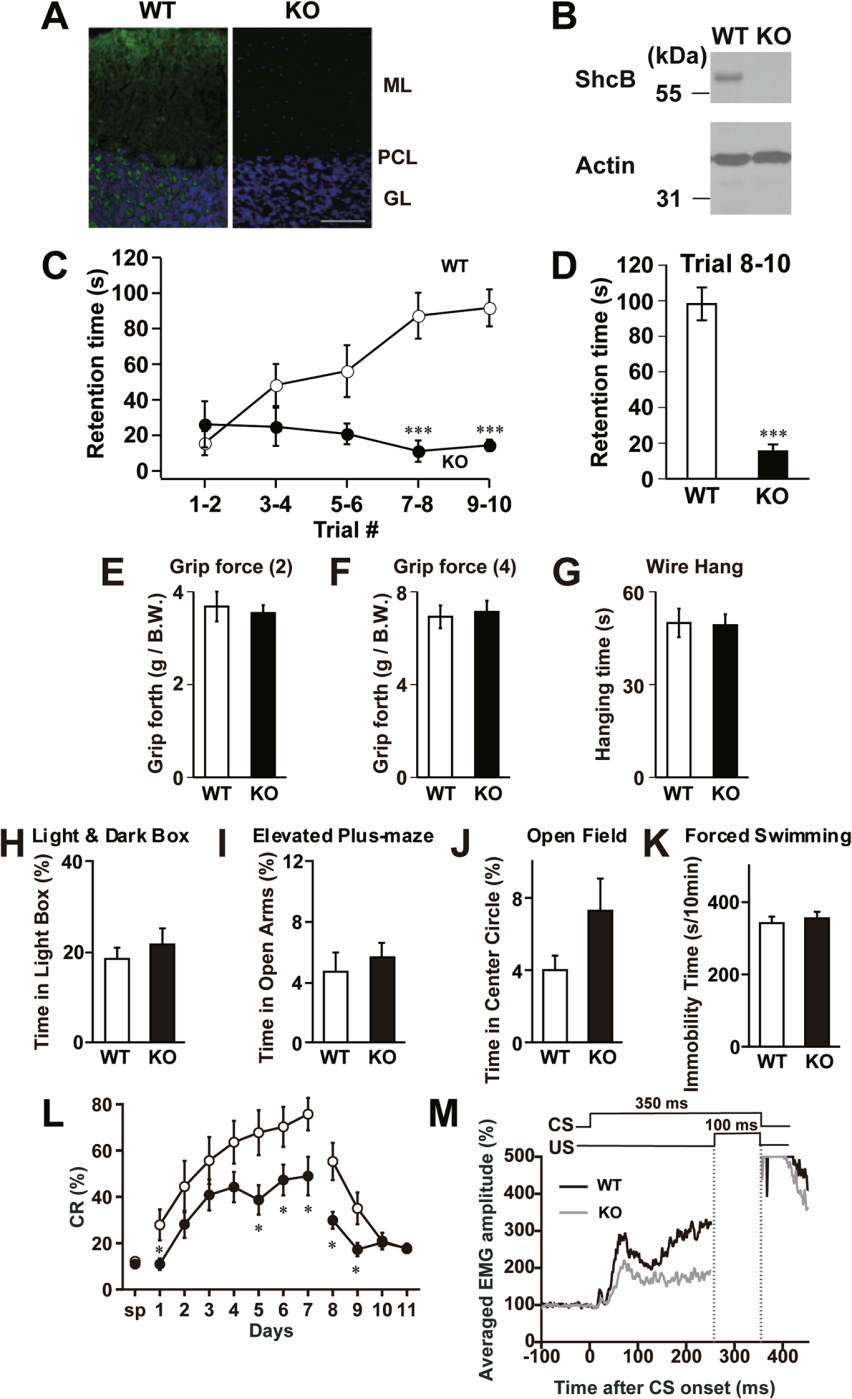
Cerebellar dysfunction in ShcB-KO mice. (**A**, **B**) Expression of ShcB protein in the cerebellum. (**A**) Immunohistochemical analysis of ShcB. ML: molecular layer, PCL: Purkinje cellular layer, GC: granular layer. Scale bar, 100 μm. (**B**) Protein expression of ShcB and actin (loading control) in the cerebellum of WT and ShcB-KO mice. Full image is shown in Fig S1A. **(C**, **D**) Retention time on rotating rod (9 rpm) of WT (n = 8) and ShcB-KO (KO; n = 6) mice. (**C**) Average values of every two trials. (**D**) Average values of trials 8–10. (**EF**) Grip force of forelimb [**E**, Grip force (2)] and both forelimb and hindlimb [**F**, Grip force (4)] of WT and ShcB-KO mice. Values are normalized by body weight (B.W). (**G**) Wire-hang test, the duration of ability to hang upside-down from a wire grid of WT (n = 13) and ShcB-KO (KO; n = 12) mice. (**H**) Light–dark box test; the time spent in the light and dark boxes was measured for 10 min of WT (n = 10) and ShcB-KO (KO; n = 10) mice. (**I**) Elevated plus-maze test; the time spent in the open and closed arms was measured for 5 min of WT (n = 10) and ShcB-KO (KO; n = 10) mice. (**J**) Open field test; the time spent in the center circle (40 cm in diameter) of the open field (120 cm in diameter) was measured for 10 min of WT (n = 10) and ShcB-KO (KO; n = 10) mice. (**K**) Forced swimming test; the immobility time in the water tank was measured every 1 min for 10 min of WT (n = 10) and ShcB-KO (KO; n = 10) mice. (**L**, **M**) (**L**) CR% and (**M**, day 7) average amplitude of eyelid EMG during delay eyeblink conditioning of WT (n = 11) and ShcB-KO (KO; n = 7) mice CR%: conditioned response frequency.

A previous study indicated predominant expression of ShcB in the cerebellar PCs of adult rats^27^. In line with this report, we observed a substantial ShcB-immunohistochemical signal in cerebellar PCs, and a weaker signal in the granular layer of adult wild-type (WT) mice but not of ShcB-knockout (ShcB-KO) mice (Fig. 1A). Biochemical assays indicated protein expression of ShcB in the WT cerebellum but not in ShcB-KO mice (Fig. 1B, Supplementary Fig. S1A). Thus, to explore the potential involvement of ShcB in the cerebellar function, we first examined motor coordination of the mice by rotor-rod test. On one hand, the retention time of WT mice on the rotating rod showed steady improvement over ten trials, whereas that of ShcB-KO mice showed less improvement and was significantly lower than that of WT mice after the 7th–8th trials (Fig. 1C,D). On the other hand, no significant differences were observed between WT and ShcB-KO mice in grip-force and wire-hang tests (Fig. 1E–G), indicating that muscle activity is normal in the ShcB-KO mice. Furthermore, the knockout mice showed no significant abnormalities in emotional-behavior tasks such as the light–dark box, elevated pulse-maze, open field and forced swimming tests (Fig. 1H–K), suggesting that susceptibility to stress in the mice was normal. We also tested the ShcB-KO mice on eyeblink conditioning, a form of cerebellum-dependent discrete motor learning, in which a tone is used as a conditioned stimulus preceding and co-terminating with an electrical shock as an unconditioned stimulus (Fig. 1M). In the acquisition trials, animals learn the adaptive timing of eyeblinking; in our study, the conditioned response frequency (CR%) for WT mice progressively and significantly increased to over 70% on day 7 (Fig. 1L). However, the CR% for ShcB-KO mice did not reach even 50% (Fig. 1L). The normalized electromyographic (EMG) amplitude on day 7 for ShcB-KO mice was also clearly lower than that for WT mice (Fig. 1M). Thus, we concluded that classical conditioning is significantly impaired in ShcB-KO mice. Because both the ability to ride a rotating rod and delayed eyeblink conditioning are cerebellum-dependent tasks^28^, we reasonably assumed cerebellar dysfunction in ShcB-KO mice.

### Impaired synaptic plasticity in the ShcB-deficient cerebellum

**Figure 2:**
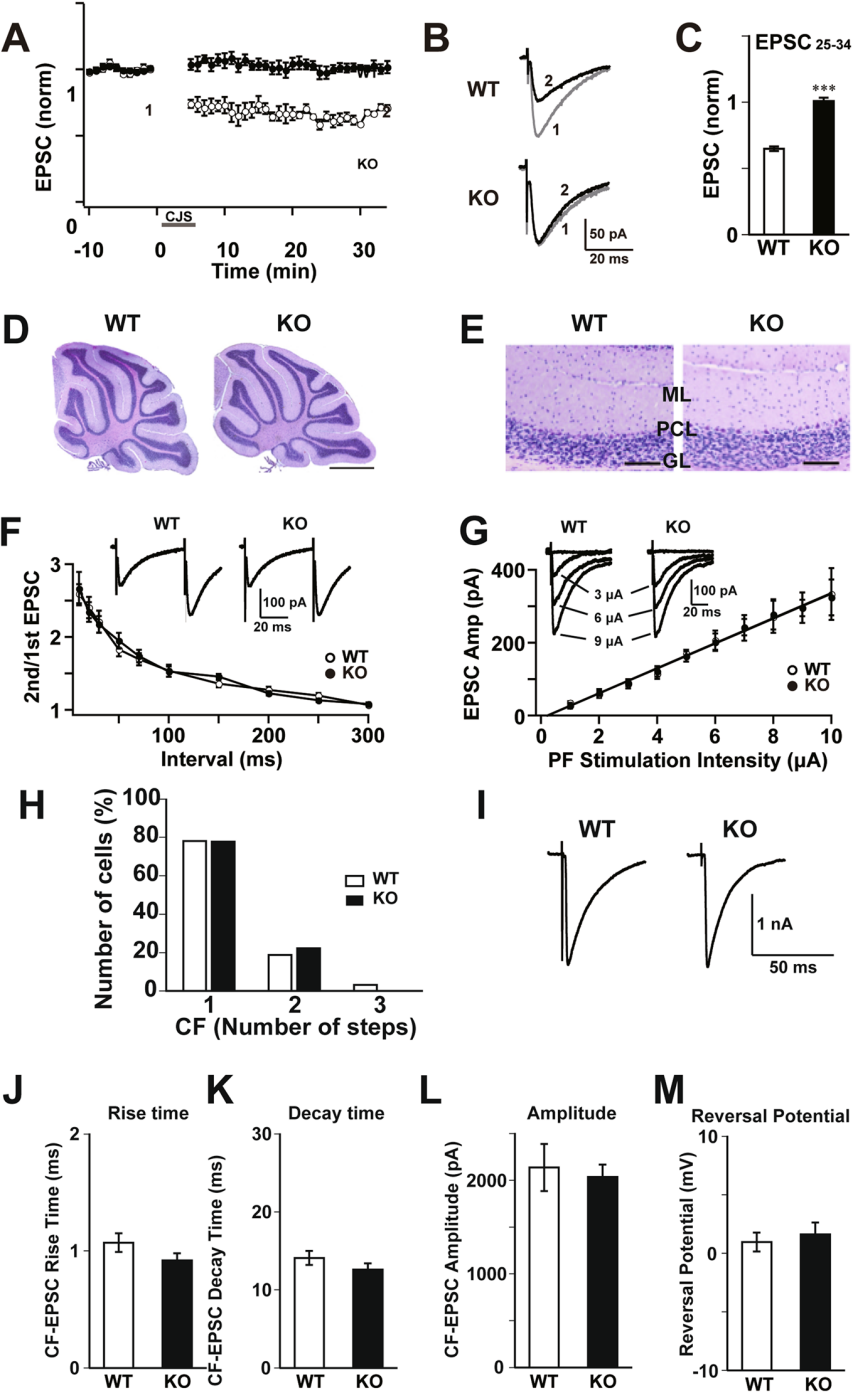
Impaired LTD in ShcB-KO cerebellum. (**A–C**) PF-PC synaptic LTD. (**A**) Amplitude of EPSC at PF-PC synapse (PF-EPSC) before and after CJS. (**B**) Typical traces of PF-EPSC (1) before and (2) 21—30 min after CJS. (**C**) The average amplitude of PF-EPSC during 21–30 min after the CJS in WT (n = 6) and ShcB-KO (KO; n = 5) mice. PF–PC: parallel fiber – Purkinje cell, LTD: long-term depression, EPSC: excitatory postsynaptic current, CJS: conjunctive stimulation. (**D**, **E**) Morphology of mature cerebellum. (**D**) Nissl staining image of the cerebellum in WT and ShcB-KO (KO) mice. Scale bar: 1 mm. (**E**) Nissl staining of cerebellar cortex in WT and ShcB-KO mice. ML: molecular layer, PCL: Purkinje cellular layer, GC: granular layer. Scale bar: 100 μm. (**F**, **G**) Basic electrophysiological properties of PF-EPSC. (**F**) Paired-pulse ratio of PF-EPSC recorded from WT (n = 6) and ShcB-KO (n = 11) PCs. Inset: Typical current responses to paired-pulse stimulation (50-ms interval). (**G**) Input–output relation of PF-EPSC. First regression lines of WT (n = 6) and ShcB-KO (n = 8) groups are shown as black and gray lines, respectively. Inset: Typical PF-EPSCs evoked by stimulation with increasing intensities. PC: Purkinje cell. (**H**, **I**) Elimination of surplus CF-PC synapse is not impaired in ShcB-KO cerebellum. (**H**) Frequency distributions of PCs in terms of the number of discrete steps of EPSC at CF-PC synapse (CF-EPSC). Open column, WT (n = 32 cells, 3 mice); closed column, ShcB-KO (n = 27 cells, 3 mice). (**I**) Typical traces of CF-EPSCs recorded from PCs in WT and ShcB-KO mice; 3–4 traces are superimposed. CF: climbing fiber, CF–PC: climbing fiber–Purkinje cell. (**J–M**) Basic electrophysiological properties of CF-EPSC in WT and ShcB-KO cerebellum. (**J**) 10–90% rise time, (**K**) decay time constant, (**L**) amplitude and (**M**) reversal potential of WT (n = 10) and ShcB-KO (KO; n = 11) mice. ***P < 0.001, compared with WT mice.

Next, we examined cerebellar long-term depression (LTD) at parallel fiber (PF)-PC synapses, because many previous studies indicate that cerebellar LTD is the cellular basis for cerebellar motor learning^29,30^. In cerebellar slices from the WT mice, the mean amplitude of PF-excitatory postsynaptic current (EPSC) 21–30 min after the LTD-inducing stimulus (conjunctive stimulus, CJS) was significantly lower than the level before CJS (Fig. 2A–C). However, in cerebellar slices from the ShcB-KO mice, the mean EPSC amplitude 21–30 min after CJS was not significantly lower than the level before CJS, but was significantly higher than that in wild-type mice (Fig. 2A–C). Thus, cerebellar LTD is impaired in ShcB-KO mice.

Cerebellar dysfunction may also result from abnormal cerebellar morphology and/or synaptic transmission. However, we found no abnormality in the morphology and basic electrophysiological properties of excitatory synapses in the mature ShcB-KO cerebellum. Cerebellar lobule formation and the three-layer structure of the cerebellar cortex were normal in ShcB-KO mice (Fig. 2D,E). Paired-pulse ratio and input–output relation and of PF-EPSC, elimination of excess climbing fiber (CF)-to-PC synapses, and basic properties of CF-synapse EPSC were not affected in the ShcB-KO cerebellum (Fig. 2F–M). These results suggest that impaired LTD does not result from abnormal formation of cerebellar morphology or impaired synaptic transmission, and that ShcB is indispensable for LTD induction at PF–PC synapses.

### Impaired Ca^2+^ release in ShcB-deficient PCs

**Figure 3:**
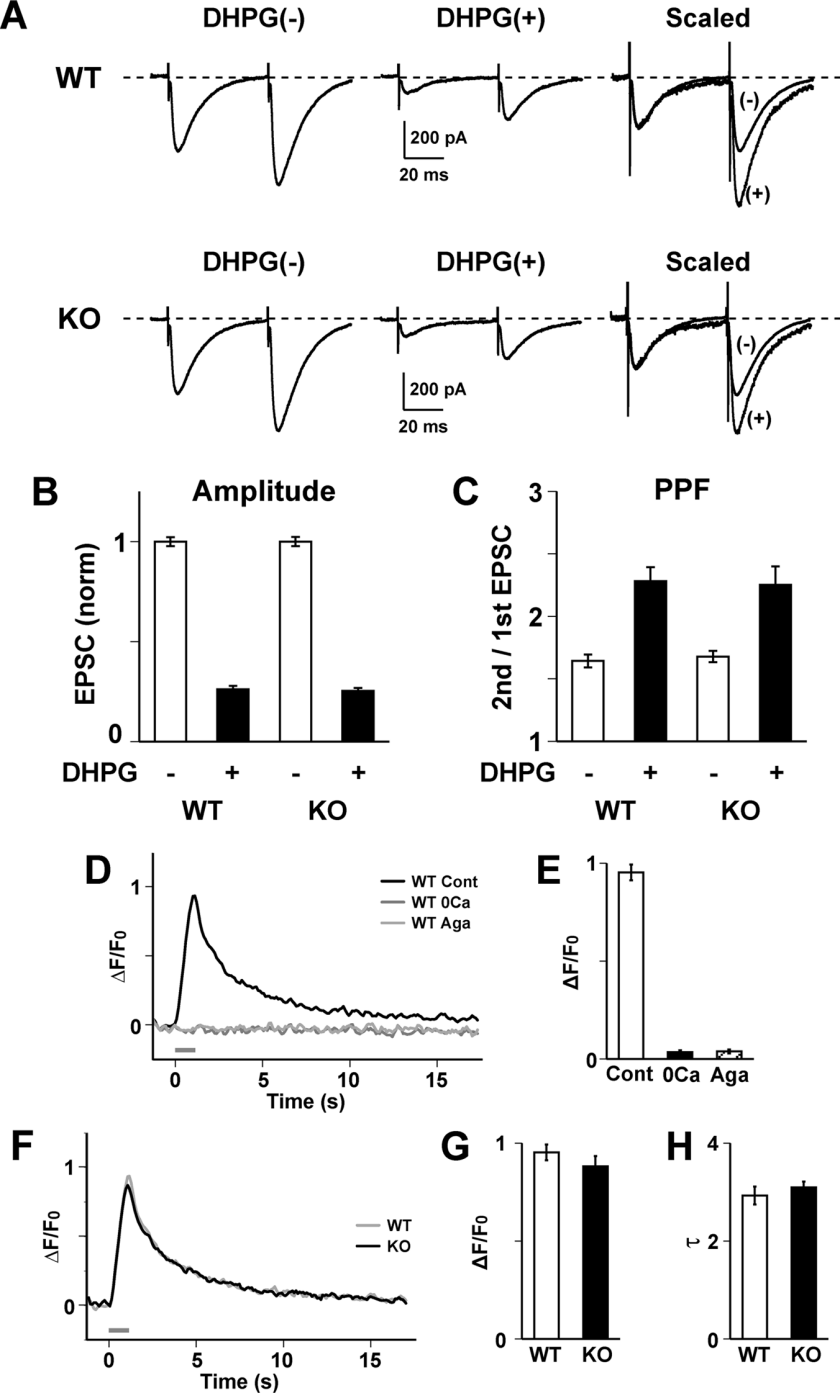
Normal function of the group 1 mGluR and voltage-dependent Ca^2+^ channels in ShcB-KO PCs. (**A**) Representative traces of PF-EPSCs recorded from WT and ShcB-KO (KO) PCs in response to paired-pulse stimuli (interpulse interval: 50 ms) before (DHPG (−)) and after (DHPG (+)) bath application of 50 μM DHPG, a specific agonist of the group 1 mGluR. PF-EPSC: parallel fiber-excitatory postsynaptic current, PC: Purkinje cell, DHPG: (S)-dihydroxyphenylglycine, mGluR: metabotropic glutamate receptor. (**B**, **C)** Decrease in (**B**) PF-EPSC amplitude by application of DHPG, accompanied by increase in (**C**) paired-pulse ratio. Amplitudes are normalized to the average before DHPG application, and are shown as mean ± s.e.m. for WT (n = 10) and ShcB-KO (KO; n = 9) mice. (**D–H)** Ca^2+^-influx through voltage-dependent Ca^2+^ channel is normal in ShcB-KO Purkinje cells (PCs). (**D**, **E**) Ca^2+^-transients elicited by depolarization-pulse (from − 80 to 0 mV for 1 s) to PCs in WT cerebellum. The response was inhibited by removing Ca^2+^ from extracellular fluid (0 Ca^2+^) or adding 200 nM ω-agatoxin (Aga). (**D**) representative image, (**E**) averaged values of amplitude. (**F–H**) Ca^2+^ influx induced by depolarization pulse to WT or ShcB-KO PCs. (**F**) Representative image, (**G**) averaged values of amplitude and (**H**) half decay time (τ) of Ca^2+^ influx of WT (n = 7) and ShcB-KO (KO; n = 7) mice. Values are normalized to those before depolarization.

Subsequently, we examined which signaling pathway is impaired in the ShcB-KO cerebellum with respect to cerebellar LTD. Metabotropic glutamate receptor 1 (mGluR1) is essential for the induction of cerebellar LTD. In cerebellar slices from WT mice, bath application of (S)-3,5-dihydroxyphenylglycine (DHPG), a selective agonist for mGluR1, decreased the PF-EPSC amplitude and increased the paired-pulse ratio, suggesting a decrease in the release probability of PF^31^ (Fig. 3A–C). DHPG induced the same effects in the ShcB-KO cerebellum and there were no significant differences between the WT and ShcB-KO cerebella, indicating that the mGluR1-mediated signaling pathway is normal in ShcB-KO PCs.

Ca^2+^ signals are also involved in the induction of various types of synaptic plasticity, including cerebellar LTD^29,32^. Application of a depolarization pulse elicited a transient increase in Ca^2+^ levels in WT PCs (Fig. 3D). Because this response is inhibited by a 0 Ca^2+^ medium or ω-agatoxin (a blocker of the P/Q-type voltage-dependent Ca^2+^ channel), the Ca^2+^ transient is indicated to be Ca^2+^ influx from the extracellular medium mediated by P/Q-type Ca^2+^ channels (Fig. 3D,E). In ShcB-KO PCs, the peak amplitude and decay time constant (τ) of the Ca^2+^ transient were not significantly different from those of WT PCs (Fig. 3F–H). Hence, Ca^2+^ influx is normal in ShcB-KO PCs. In addition, because the amplitude of Ca^2+^ influx is determined by Ca^2+^ levels in the extracellular medium and cytoplasm, this result also indicates that intracellular Ca^2+^ level is not affected in ShcB-deficient PCs.

**Figure 4:**
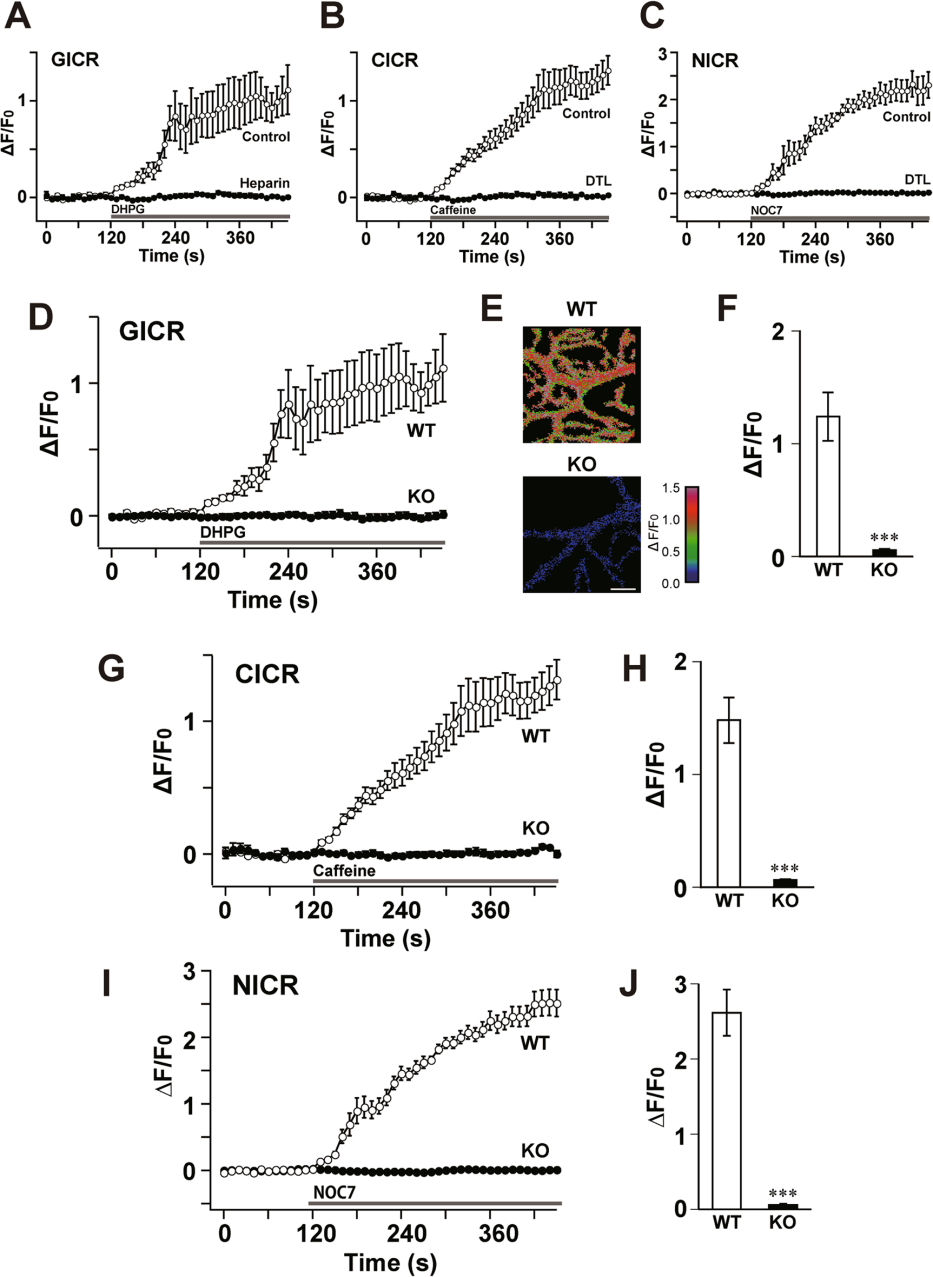
Impaired Ca^2+^ release in ShcB-KO PCs. (**A–C**) Ca^2+^ release induced by bath application of (**A**) DHPG, (**B**) caffeine or (**C**) NOC7 in the presence or absence or (**A**) heparin or (**B**, **C**) dantrolene. (**D–J**) (**D–F**) DHPG—(GICR; WT (n = 7) and KO (n = 14)), (**G**, **H**) caffeine- (CICR; WT (n = 10) and KO (n = 10)) and (**I**-**J**) NOC7-induced (NICR; WT (n = 5) and KO (n = 7)) Ca^2+^ elevation. (**A**, **D**, **F**) Intracellular Ca^2+^ levels before and after bath application of 50 µM DHPG, 25 mM caffeine and 300 μM NOC7. (**E**) Pseudocolor Ca^2+^ images of PCs 330 s after DHPG application. Scale bar: 10 μm. (**F**, **H**, **J**) Peak value of GICR (**D**), CICR (**G**) and NICR (**I**). Values are normalized to those before drug application. ***P < 0.001, significantly different from the value for control group. GICR: glutamate-induced Ca^2+^ release, CICR: caffeine-induced Ca^2+^ release, NICR: nitric oxide-induced Ca^2+^ release, PC: Purkinje cell.

Ca^2+^ release from intracellular stores is also necessary for LTD induction^33,34^. Bath application of DHPG (mGluR1 agonist) induced a prominent increase in Ca^2+^ levels in WT PCs (Fig. 4A,D–F). This Ca^2+^ elevation was mediated by Ca^2+^ release through the inositol-1,4,5-phosphate receptor (IP_3_R) (glutamate-induced Ca^2+^ release; GICR), because pipette application of heparin (4 mg/ml), an IP_3_R blocker, abolished the DHPG-induced Ca^2+^ elevation (Fig. 4A). However, DHPG did not induce any Ca^2+^ elevation in ShcB-KO PCs (Fig. 4D–F). Because the application of DHPG induced a decrease in PF-EPSC and an increase in the paired-pulse ratio in ShcB-KO PCs (Fig. 3A–C), the impaired GICR in ShcB-KO PCs was not due to the functional abnormality of mGluR-phospholipase C (PLC) signaling in ShcB-KO PCs. Therefore, we interpret this to mean that IP_3_R-mediated Ca^2+^ release is impaired in ShcB-KO PCs.

We also examined Ca^2+^ release through ryanodine receptors (RyRs) in PCs. Bath application of caffeine, a broad agonist for RyRs, induced a clear increase in intracellular Ca^2+^ levels in WT PCs (Fig. 4B,G,H). This Ca^2+^ elevation was confirmed to be mediated by RyR, because dantrolene, a specific antagonist for RyR, completely abolished caffeine-induced Ca^2+^ elevation in PCs (Fig. 4B). However, the caffeine-induced Ca^2+^ release (CICR) was diminished in ShcB-KO PCs (Fig. 4G,H). Another type of RyR-mediated Ca^2+^ release, nitric oxide-induced Ca^2+^ release (NICR)^35–37^, is also observed in cerebellar PCs (Fig. 4C,I,J). NICR is elicited by S-nitrosylation of type 1 ryanodine receptor (RyR1) and blocked by dantrolene (Fig. 4C). This response was again impaired in ShcB-KO PCs (Fig. 4I,J). Taken together, we demonstrated that Ca^2+^ release through both IP_3_R and RyR1 is abolished in the cerebellar PCs of ShcB-KO mice.

### Dysfunction of ER Ca^2+^ store in ShcB-deficient PCs

**Figure 5:**
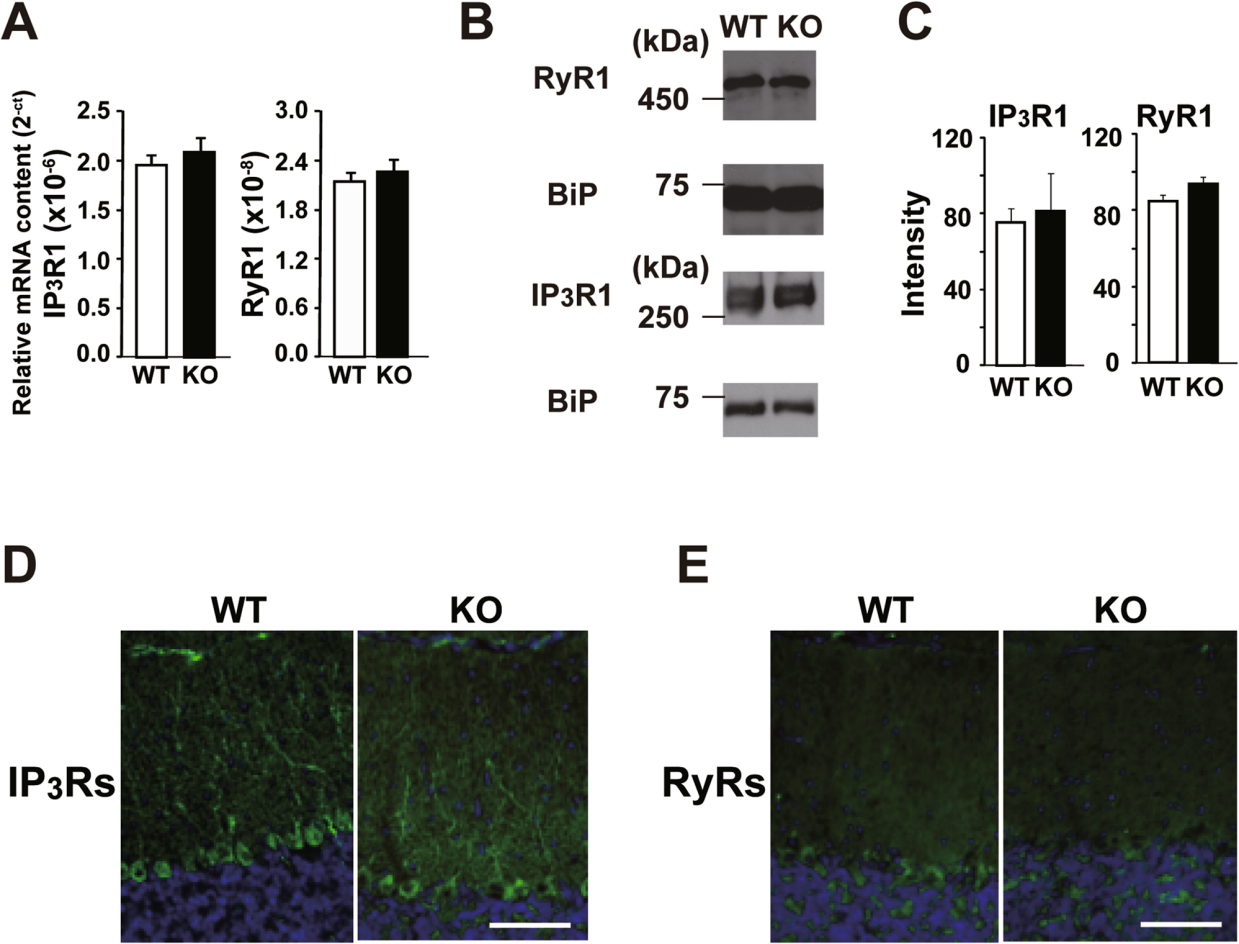
Normal expression of Ca^2+^ release channels in ShcB-KO PCs. (**A**) Relative mRNA content of IP_3_R1 and RyR1, analyzed with real-time PCR in WT (n = 5) and ShcB-KO (KO; n = 5) mice. IP_3_R1: type 1 inositol-1,4,5-phosphate receptor, RyR1: type 1 ryanodine receptor. (**B**, **C**) (**B**) Protein expression of IP_3_R1, RyR1 and BiP (loading control). Full images are shown in Fig S1B,C. (**C**) Signal intensities in immunoblot in WT (n = 3) and ShcB-KO (KO; n = 3) mice. Bip: immunoglobulin heavy chain-binding protein. (**D**, **E**) Intracellular distribution of (**D**) IP3Rs and (**E**) RyRs. Scale bar: 50 μm.

These defects in the Ca^2+^ release have several possible underlying mechanisms. The expression and/or localization of Ca^2+^ release channels could be disrupted in ShcB-KO PCs. Of all Ca^2+^ release channel subtypes, type 1 IP_3_R (IP_3_R1) and RyR1 are the most dominant subtypes expressed in PCs^38,39^, and are essential for the induction of cerebellar LTD^33,34^. However, there were no significant differences in relative mRNA content (Fig. 5A) and protein levels (Fig. 5B,C and Supplementary Fig. S1B,C) of IP_3_R1 and RyR1 between ShcB-KO and WT cerebella. In addition, immunohistochemical analysis using antisera for IP_3_Rs and RyRs detected no prominent differences in the expression of IP_3_Rs and RyRs, respectively, between WT and ShcB-KO PCs (Fig. 5D,E).

**Figure 6:**
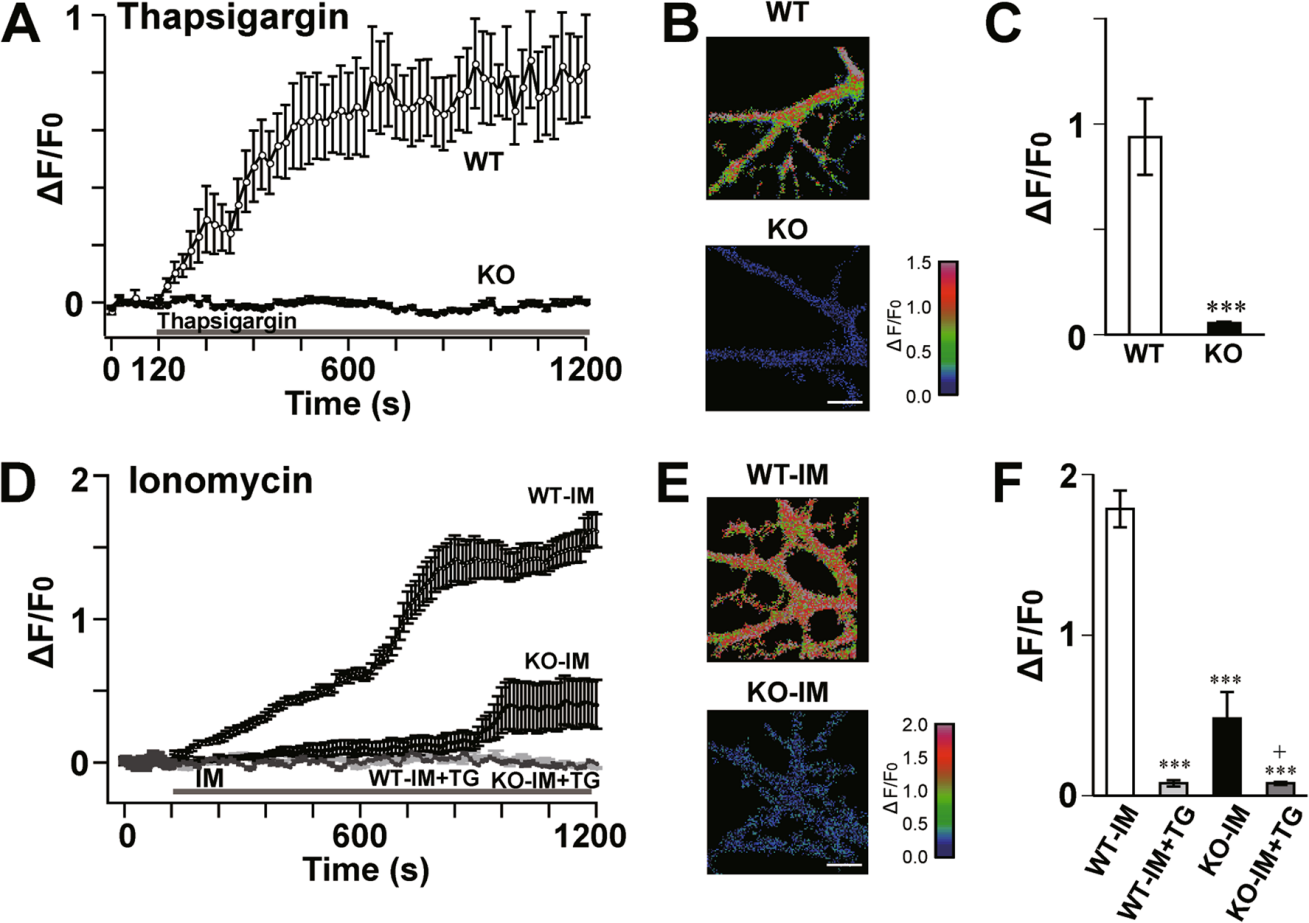
Decreased Ca^2+^-store content and SERCA activity in ShcB-KO PCs. (**A–F**) (**A–C**) Effects of 2 μM TG or (**D–F**) 2 μM IM on Ca^2+^ levels in PCs. (**A**, **D**) Ca^2+^ levels before and after bath application of the drug. n ≥ 7. (**B**, **E**) Pseudocolor Ca^2+^ images of PCs 18 min after the beginning of drug application. (**C**, **F**) Averaged peak value of fluorescent signals. ***P < 0.001, compared with WT-IM, and ^+^*P* < 0.05, compared with KO-IM. TG: thapsigargin, IM: ionomycin, PC: Purkinje cell.

We then examined the possibility of dysfunction of Ca^2+^ stores in ShcB-KO PCs. In the resting condition, spontaneous Ca^2+^ release from intracellular stores and Ca^2+^ uptake by SERCA and plasma-membrane Ca^2+^-ATPase (PMCA) are balanced, and the intracellular Ca^2+^ levels are kept constant. Thus, in WT PCs, acute inhibition of SERCA by a specific inhibitor, thapsigargin, results in a continuous increase in intracellular Ca^2+^ levels (Fig. 6A–C) because the spontaneous Ca^2+^ release surpasses the uptake^40^. This thapsigargin-induced Ca^2+^ elevation was largely decreased in ShcB-KO PCs (Fig. 6A–C), suggesting that the Ca^2+^ stores and SERCA activity are diminished in ShcB-KO PCs. To further confirm this hypothesis, we also examined the effects of ionomycin, a calcium ionophore, on cytosolic Ca^2+^ levels in PCs. When ionomycin is applied at 1–2 µM, the drug is incorporated into the ER membrane selectively, and induces Ca^2+^ leakage from intracellular stores. After bath application of 2 µM ionomycin, a slow but clear increase in intracellular Ca^2+^ level was observed in WT PCs when using Ca^2+^-free extracellular fluid (Fig. 6D–F). This elevation was confirmed to arise from Ca^2+^ release from intracellular stores, because pretreatment with 2 µM thapsigargin for 60 min almost completely blocked the ionomycin-induced Ca^2+^ elevation (Fig. 6D,F). In ShcB-KO PCs, however, the ionomycin–induced Ca^2+^ leak was significantly and greatly reduced. Because this response was almost completely inhibited by pretreatment with thapsigargin (Fig. 6F), the result strongly suggested that the contents of Ca^2+^ stores are decreased in ShcB-KO PCs.

### SERCA activity reduction in the ShcB-KO cerebellum

**Figure 7:**
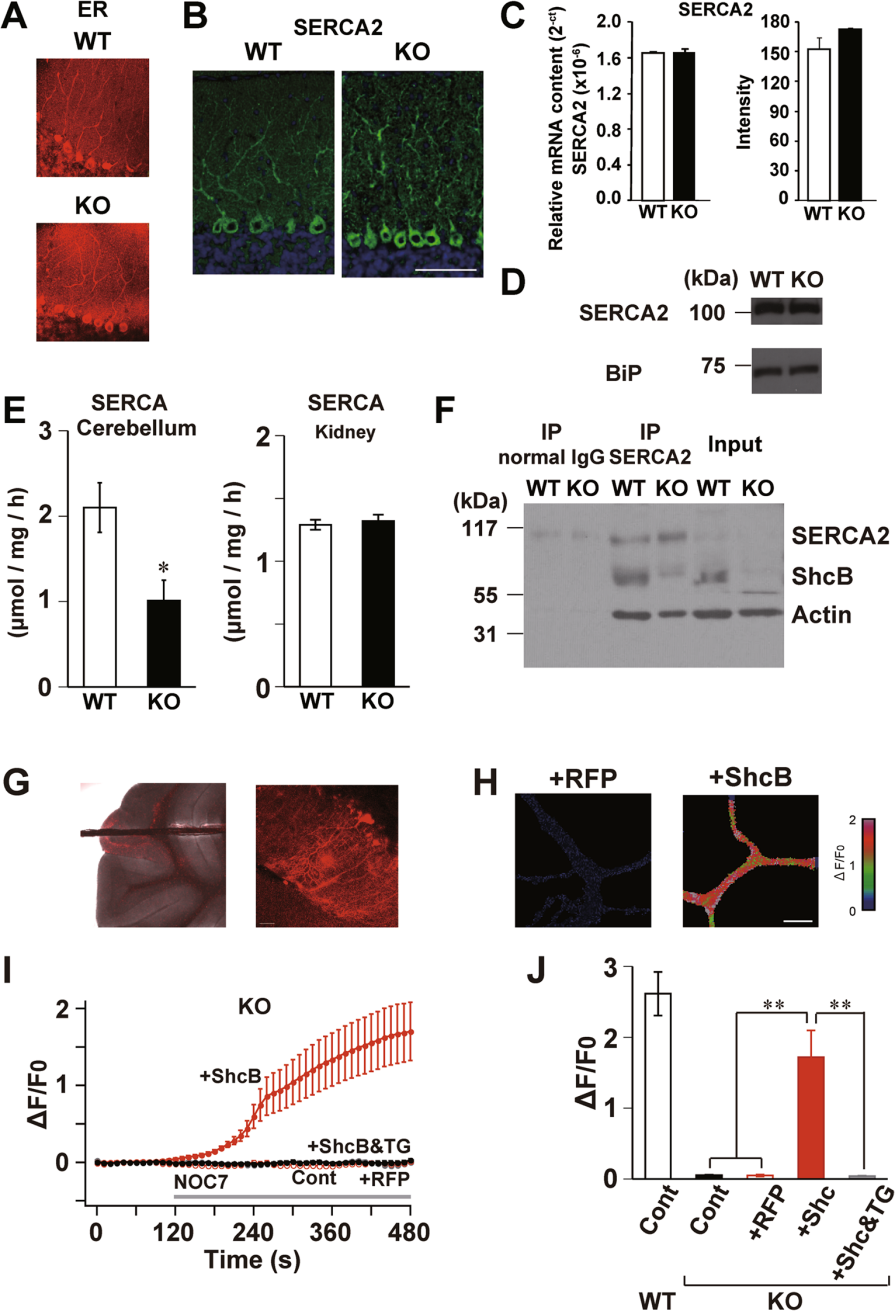
Homeostatic regulation of Ca^2+^-store content by ShcB through the regulation of SERCA activity. (**A**, **B**) Intracellular distribution of (**A**) rER and (**B**) SERCA2 protein. Scale bar: 50 μm. rER: rough endoplasmic reticulum, SERCA: sarco/endoplasmic reticular Ca^2+^-ATPase. (**C**, **D**) Expression of SERCA2 and BiP (loading control). Full image is shown in Fig S1D. (**C**) Relative mRNA content (left) and protein level (right) in WT (n = 5) and ShcB-KO (n = 3) mice. (**D**) Representative data of immunoblot analysis. (**E**) SERCA activity in lysate of cerebellum from WT (n = 12) and ShcB-KO (KO; n = 7) mice. (**F**) Western-blot signals detected by anti-SERCA2 antiserum or anti-ShcB antiserum after IP by mouse anti-SERCA2 antiserum (IP: SERCA2) or by normal mouse IgG (IP: normal IgG). Lysates from WT and ShcB-KO cerebellum without IP (Input) were also applied. IP: immunoprecipitation. Full image is shown in Fig S1E. (**G–J**) Restored Ca^2+^-store content and SERCA activity in ShcB-KO PCs by ectopic expression of ShcB (**G**) Fluorescent images of ShcB-KO cerebellum (left) and PC (right) infected with mRFP and ShcB. (**H**) Pseudocolor Ca^2+^ images of ShcB-KO PCs infected with mRFP (+RFP) or mRFP and ShcB (+ShcB) 6 min after NO application. Scale bar: 10 μm. (**I**, **J**) (**I**) Time-course and (**J**) peak amplitude of NICR in WT PC (WT Cont in D; n = 5) and ShcB-KO PC (KO) with no infection (KO Cont; n = 7) or infected with mRFP only (+RFP; n = 10) or mRFP and ShcB (+ Shc; n = 6). The recovered NICR was blocked by 2 μM TG (+Shc&TG; n = 5). Values are normalized to those before drug application. **P < 0.01, compared with the control in the ShcB-KO group. PC: Purkinje cell, NO: nitric oxide, NICR: nitric oxide-induced Ca^2+^ release, TG: thapsigargin.

We next investigated possible mechanisms behind the decreased Ca^2+^-store content in ShcB-KO PCs. First, the distribution of the rough endoplasmic reticulum (rER), a ubiquitous store of Ca^2+^ in various types of cells, was analyzed using an ER tracker (Fig. 7A). We found no clear differences in the signal derived from the ER tracker between WT and ShcB-KO PCs. Activity of SERCA is essential for Ca^2+^ uptake from the cytosol to the ER lumen^41^. Of the three types of SERCA, designated SERCA1-3, cerebellar PCs mainly express SERCA2^42^ and therefore, we analyzed the expression and distribution of SERCA2. Immunohistochemical analyses showed no prominent differences in the SERCA2 signal in PCs between WT and ShcB-KO mice (Fig. 7B). In addition, the relative mRNA content (Fig. 7C) and protein levels of SERCA2 (Fig. 7D, Supplementary Fig. S1D) were not significantly changed in the ShcB-KO cerebellum. Collectively, these findings indicate that the intracellular distribution of ER and expression of SERCA are essentially normal in ShcB-KO PCs.

We further biochemically analyzed the activity of SERCA in cerebellar lysates using an ATPase assay. Relative to the WT cerebellum, SERCA activity was significantly decreased in the ShcB-KO cerebellum (Fig. 7E). On the other hand, there was no significant difference in SERCA activity in the kidney, a tissue in which ShcB expression is not reported and was therefore used as a negative control^19^. Moreover, an immunoprecipitation experiment demonstrated possible binding of SERCA2 to ShcB in the cerebellum (Fig. 7F, Supplementary Fig. S1E). These results indicate ShcB-dependent regulation of SERCA activity in the cerebellum.

### Restoration of ER Ca^2+^ content by ectopic expression in ShcB-deficient PCs

Finally, to test whether ectopic expression of ShcB can restore the Ca^2+^-store content in ShcB-KO PCs, recombinant lentiviral transgenes for both ShcB and monomeric red fluorescence protein (mRFP) or only mRFP were injected into the cerebellar cortex of the mutant mice. Approximately 8 weeks later, Ca^2+^ release was analyzed from PCs in a virus-injected area of ShcB-KO cerebellar slices to examine functional recovery of ER Ca^2+^ store (Fig. 7G). NICR was examined because it is the most prominent of the three types of Ca^2+^ release observed in PCs (GICR, CICR and NICR). In non-infected PCs or PCs expressing only mRFP, bath application of nitric oxide (NO) did not elicit Ca^2+^ elevation (Fig. 7H–J). On the other hand, in PCs expressing both mRFP and ShcB, NO-induced Ca^2+^ elevation was clearly seen (Fig. 7H–J). We confirmed that this Ca^2+^ elevation arose from NICR as we were able to almost completely abolish the response by pretreating the slice with thapsigargin (Fig. 7I,J). The result also indicated that SERCA activity was restored in PCs with ectopic expression of ShcB. Hence, in cerebellar PCs, the ShcB-mediated signal is necessary for homeostatic regulation of ER Ca^2+^ stores, possibly through regulation of SERCA activity.

## Discussion

The present study demonstrated that the contents of intracellular Ca^2+^ store are largely decreased in ShcB-KO cerebellar PCs. Although the expression level of SERCA2, a Ca^2+^-ATPase expressed in the ER membrane, was not significantly reduced in ShcB-KO cerebella, SERCA-dependent components of Ca^2+^ release were severely impaired in ShcB-KO PCs. In addition, a decrease in SERCA activity in ShcB-KO cerebellum was also observed using biochemical analysis. These results suggest that SERCA activity is decreased in ShcB-KO PCs. Furthermore, ectopic expression of ShcB in ShcB-KO PCs rescued the SERCA-dependent component of Ca^2+^ release. Cumulatively, these observations support the involvement of ShcB in the homeostatic regulation of Ca^2+^ stores in cerebellar PCs through the regulation of SERCA activity. Thus far, four Shc-family adaptor proteins have been reported: ShcA, ShcB, ShcC and ShcD. However, there have been no studies suggesting a role for Shc-family proteins in Ca^2+^ signaling in CNS neurons or in other tissues^13^. Therefore, the present study is the first to indicate an essential role of Shc-family proteins in intracellular Ca^2+^ signaling, especially in homeostatic regulation of intracellular Ca^2+^ stores.

In the ShcB-KO cerebellum, the ER Ca^2+^ store content was largely decreased in PCs (Fig. 6D–F), whereas SERCA activity was decreased by about 50% (Fig. 7E). Therefore, there is a discrepancy in the decrease in ER Ca^2+^ store content and degree of change in SERCA activity in the ShcB-KO cerebellum, which might be due to spontaneous Ca^2+^ release in PCs. As is seen in Fig. 6A, application of thapsigargin, a SERCA inhibitor, clearly increases the Ca^2+^ levels in WT PCs. Because any other stimulus was not applied during this experiment, the result indicates that a substantial degree of spontaneous Ca^2+^ release occurs through IP_3_Rs, RyRs or unidentified Ca^2+^-leak channel(s) in PCs. When SERCA activity is not impaired, the spontaneous Ca^2+^ release is balanced by Ca^2+^ uptake by SERCA and the content of ER Ca^2+^ store is maintained homeostatically. However, in case where SERCA activity is reduced to about 50% of the normal level, spontaneous Ca^2+^ release might exceed Ca^2+^ uptake by SERCA, depleting the Ca^2+^ stores.

Regulation of SERCA activity by binding with other proteins has been previously reported^43,44^. In the heart, it is well-established that the activity of SERCA2a is regulated by phospholamban (PLN) in the cardiac ER^45^. When the cytosolic Ca^2+^ concentration is low, dephosphorylated PLN interacts with SERCA2a and inhibits pump activity, but this inhibitory effect is alleviated when PLN is phosphorylated as a downstream effect of high cytosolic Ca^2+^ concentrations. Sarcolipin (SLN), a homologue of PLN, is also well known to bind and inhibit SERCA2a in skeletal muscle^46^. Because the expression of PLN or SLN in CNS neurons has not been previously reported, the protein(s) regulating SERCA activity in these neurons are yet to be determined. In the present study, however, the SERCA-dependent component of Ca^2+^ release was impaired in ShcB-KO PCs and rescued by ectopic expression of ShcB (Fig. 7H-J). In addition, immunoprecipitation analysis demonstrated binding of ShcB with SERCA2 (Fig. 7F). These results suggest that ShcB binds to SERCA and regulates its activity in neurons, as PLN and SLN are in cardiac and skeletal muscle cells, respectively. The binding of IRS-1, IRS-2 or α-fodrin are also possibly involved in this process^10,11^. IRS proteins contain 8–18 potential tyrosine phosphorylation sites, which upon phosphorylation mediate binding to effector proteins, typically to their SH2 domains^47^. Studies on muscle and β-cells demonstrate that IRS-1 and IRS-2 bind to SERCA and that this binding is stimulated by treatment of the cells with insulin, which induces phosphorylation of IRS proteins. There are at least three tyrosine phosphorylation sites in the CH1 region of the ShcB protein, and these sites are phosphorylated upon stimulation^26^. Although identification of the binding site of ShcB to SERCA2 requires further studies, these observations suggest that ShcB binds to SERCA2 through its tyrosine phosphorylation sites, and contributes to the maintenance of ER Ca^2+^ levels by regulating SERCA activity.

Of the four Shc-family proteins, expression of ShcB and ShcC is dominant in the brains of adult mice, whereas ShcA expression gradually decreases during postnatal development^15–17^. Functional roles of ShcB were examined in a previous study using ShcB-KO mice, and a loss of peptidergic and nonpeptidergic nociceptive sensory neurons were revealed in ShcB-KO mice^17^. Therefore, it is possible that ShcB is involved in the development and maturation of nociceptive neurons through regulation of intracellular Ca^2+^ stores, although a relationship between Ca^2+^ release and development and/or maturation of sensory neurons is yet to be established. In the present study, eyeblink conditioning, a type of cerebellar-dependent motor learning, and LTD at PF synapses were impaired in ShcB-KO mice. Many studies have indicated that cerebellar LTD is the cellular basis for cerebellar-dependent motor learning, and that Ca^2+^ signals including Ca^2+^ release from intracellular store in PCs are essential for plasticity induction^48,49^. Therefore, we propose that ShcB has an essential role in cerebellar-dependent learning and cerebellar synaptic plasticity through the homeostatic regulation of intracellular Ca^2+^ stores.

In conclusion, the present study reveals that ShcB-mediated signaling has critical role in homeostatic maintenance of Ca^2+^ store in PCs. Future studies may illuminate the involvement of other Shc-family and related molecules in Ca^2+^-store regulation in neuronal cells other than PCs as well as in non-neuronal cells. The ER is a dynamic intracellular store of Ca^2+^ that regulates cytosolic Ca^2+^ concentrations, and the indispensable role of ER Ca^2+^ stores in various cellular function have been extensively demonstrated in various excitable and non-excitable cells. ER Ca^2+^ homeostasis is essential for protein synthesis, cell differentiation and cell survival; in the CNS, perturbations in ER-mediated Ca^2+^ regulation possibly contribute to neurodegenerative processes. Our study sheds light on ShcB as a novel signaling molecule involved in homeostatic regulation of intracellular Ca^2+^ stores, and presents its therapeutic potential for diseases resulting from dysfunction of ER Ca^2+^ homeostasis.

## Materials and methods

### Animals experiments

All experiments were carried out according to the guidelines and approved by the Animal Welfare Committees of Nagasaki University, Kyoto University, Tokushima Bunri University, University of Toyama, Hokkaido University and Kitasato University. Efforts were made to minimize animal suffering and to reduce the number of animals used. WT and ShcB-KO mice at 8–12 weeks of age were used for all analyses. ShcB-KO mice were generated as described previously^17^. The mice were housed in plastic cages and kept in a regulated environment (24 ± 1 °C; 50 ± 5% humidity), with a 12 h light/dark cycle (lights on at 8:00 A.M.). Food and tap water were available ad libitum.

### Histological analyses

Mice were pentobarbital-anesthetized and perfused with 4% paraformaldehyde in phosphate-buffered saline. Then, the brains were removed, postfixed for 24 h in 4% paraformaldehyde, and cryoprotected in 30% sucrose. Cerebellar sections (15 µm) were cut, mounted on slides and processed for Nissl staining or immunohistochemical staining. Staining with hematoxylin and Nissl staining were performed according to standard procedures. For immunochemical staining, the sections were permeabilized with 0.2% Triton X-100, blocked with 5% BSA, incubated with antibody overnight at 4 °C, and incubated further with Alexa Fluor 488-conjugated secondary antibody for 1 h at 37 °C. Antibody against ShcB was prepared as described previously^17^. Antibodies against IP_3_Rs (sc-28613), RyRs (sc-8170) and SERCA2 (sc-8095) were purchased from Santa Cruz Biotechnology (Dallas, TX, USA). Fluorescence images were obtained with the confocal imaging system Micro Radiance (Bio-Rad Laboratories, Hercules, CA, USA).

### Rotor-rod analysis

In the rotor-rod test, as with other behavioral tests including eyeblink conditioning and those described in supplemental information, 8- to 12-week-old WT- and ShcB-KO mice were used. The ability for motor coordination was examined with rotor-rod analysis^50,51^. Mice were placed on the stationary rod (diameter = 9 cm) of a rotating-rod device (Muromachi Kikai, Tokyo, Japan) for up to 120 s until they habituated to the experimental environment. The mice were then carefully placed on the rotating rod (9 rpm), and the time they remained on the rod was measured for each trial. The maximum retention time was 120 s.

### Eyeblink conditioning

Eyeblink conditioning was carried out as described previously^34,52^. For delay eyeblink conditioning, mice were surgically implanted with four Teflon-coated stainless-steel wires (A-M Systems, WA, USA) under the left eyelid for recording EMG activity and delivering electric shock. A periorbital shock (100 Hz square pulses for 100 ms) was applied as unconditioned stimulus, and a tone (1.0 kHz, 80 dB for 350 ms) was used as the conditioning stimulus; the conditioning stimulus preceded and co-terminated with the unconditioned stimulus (see Fig. 1M). After spontaneous eyeblink frequency (sp) was examined, the mice were subjected to the acquisition session for 7 days and the extinction session for 4 days. The acquisition session consisted of 100 paired trials of the conditioned and unconditioned stimuli. The extinction sessions consisted of 100 conditioned stimulus trials. The spontaneous eyeblink frequency was measured in 100 non-stimulus trials. The electromyographic signal was band-pass filtered between 0.15 and 1.0 kHz and fed into a computer at a sampling rate of 10 kHz. The EMGs were analyzed as described previously^53^. A threshold was determined, and the time window selected for evaluating the CR was 100 ms before US onset. The time window was extended by 100 ms to obtain the CR value in the CS-only trials. As shown in Fig. 1E, the accumulated amplitude in the ShcB-KO mice was significantly lower than that in the WT mice due to decreased CR%. The transient electromyographic response at ~ 20 ms preceding the CR at ~ 80–350 ms was derived from the auditory startle reflex elicited upon the sound and was retained in the ShcB-KO mice. Therefore, it is unlikely that the poor performance is caused by hearing inability in the ShcB-KO mice.

### Grip force test

In grip force test, as well as other behavioral tests described below, 8- to 12-week-old WT- and ShcB-KO mice were used. Mice were gently held by the tail and allowed them to grasp the horizontally positioned metal bar of the Grip Strength Meter (MK308M, Muromachi Kikai, Tokyo, Japan) with their forelimbs (2-grips test) or forelimbs and hindlimbs (4-grips test). Each mouse performed three trials for force measurement. The highest force value applied to the metal bar was recorded^54^.

### Grid-hang test

Each mouse was placed on the center of a 0.5 cm grid at the bottom of a 28 cm × 36 cm box. The mouse was supported by the experimenter until it grabbed the grid with both its fore- and hind-paws. The grid was then inverted so the mouse was hanging upside-down. The duration of time that the animal remained on the grid was recorded. Only mice capable of hanging onto the grid for at least 1 s were included in analysis^55^. The time to falloff, with a 60-s cutoff time, was measured to evaluate the prehensile reflex. Three trials were performed for each mouse with a 20-min interval.

### Light and dark box test

In light and dark box test, as well as other behavioral tests described below, WT- and ShcB-KO mice at 12-week old were used. The light and dark box consisted of two compartments: a transparent Plexiglas box with a white Plexiglas floor and a black Plexiglas box with a black Plexiglas floor (both 15 × 15 × 15 cm)^56^. Each box could be divided by a sliding door (10 × 5 cm high). The test was started by placing a mouse in the black Plexiglas box. The time spent in the transparent and black Plexiglas boxes was measured for 10 min using digital counters with infrared sensors (Scanet SV-10 LD; TOYOSANGYO, Yasu, Japan). The data were used to calculate the percentage of time spent in the light box: (time spent in transparent Plexiglas box/time spent in transparent and black Plexiglas boxes) × 100.

### Elevated plus-maze test

The elevated plus-maze consisted of two open arms (25 × 8 × 0.5 cm) and two closed arms (25 × 8 × 20 cm) emanating from a central platform (8 × 8 cm) to form a plus shape. The entire apparatus was elevated at a height of 50 cm above floor level. The test was started by placing a mouse on the central platform of the apparatus facing an open arm. The time spent in the open and closed arms was measured manually for 5 min. The data were used to calculate the percentage of time spent in the open arms: (time spent in open arms/ time spent in open and closed arms) × 100^56^.

### Open field test

The open field test was started by placing a mouse at the edge of the wall of a circular open field (120 cm in diameter × 25 cm high)^16^. Mouse was allowed to freely explore for 10 min, its path of movement tracked with a video camera fixed on the ceiling of the room and stored in a computer system (AXIS-90 Target/2; Neuroscience). The time spent in a concentric circular area (40 cm in diameter) of the open field was analyzed using the stored data.

### Forced swimming test

In the forced swimming test, a mouse was placed in a transparent glass cylinder (8 cm in diameter × 20 cm high), which contained water at 25 °C at a depth of 8 cm, and was forced to swim for 10 min. The duration of immobility was measured every 1 min using digital counters with infrared sensors (Scanet MV-10 AQ; TOYOSANGYO), as described previously^57^.

### Slice preparation and whole-cell patch-clamp recording

WT and ShcB-KO mice were sacrificed by cervical dislocation under deep anesthesia with diethyl ether. The cerebellum was excised, and parasagittal cerebellar slices (250 μm thick) were prepared from the vermis^50,51,58^. Whole-cell recordings were obtained from visually identified PCs under an upright microscope (BX51WI, OLYMPUS, Tokyo, Japan) using a 40 × water-immersion objective at room temperature (23–25 °C), except for the experiments using dantrolene (36 ± 1 °C). The resistance of patch pipettes was 2.5–4.0 MΩ when filled with an intracellular solution composed of (in mM) 120 K-gluconate, 5 KCl, 5 NaCl, 1 EGTA, 4 ATP, 0.4 GTP and 10 HEPES (pH 7.3; adjusted with KOH). For the voltage-clamp recording of CF-EPSCs, a pipette solution with the following composition was used (in mM) 60 CsCl, 10 Cs D-gluconate, 20 TEA-Cl, 20 BAPTA, 4 MgCl2, 4 ATP, 0.4 GTP and 30 HEPES (pH 7.3). The standard bathing solution was composed of (in mM) 125 NaCl, 2.5 KCl, 2 CaCl_2_, 1 MgSO_4_, 1.25 NaH_2_PO_4_, 26 NaHCO_3_ and 20 glucose, which was bubbled continuously with a mixture of 95% O_2_ and 5% CO_2_. Bicuculline (10 μM) was always present in the saline to block spontaneous inhibitory postsynaptic currents. Square pulses were applied for focal stimulation (duration, 0.1 ms; amplitude, 0–90 V for CF stimulation, 0–10 V for PF stimulation) through a glass pipette with a 5 to 10 μm-diameter tip and filled with the standard bath solution. The membrane potentials were held at − 90 to − 80 mV for recording PF-EPSCs, and at − 20 to − 10 mV for recording CF-EPSCs, after the compensation for liquid junction potential. Ionic currents were recorded using a patch-clamp amplifier (EPC-9, HEKA, Lambrecht/Pfalz, Germany). Stimulation and on-line data acquisition were performed using PULSE software (HEKA) on a Macintosh computer. Signals were filtered at 3 kHz and digitized at 20 kHz. The fitting of the decay phases of EPSCs was performed with PULSE-FIT software (HEKA).

For the LTD experiments, the intensity of the stimulus was adjusted to evoke PF-EPSCs whose initial amplitudes were between 80 and 150 pA. After obtaining a stable initial recording for at least 10 min, a conjunctive stimulus was applied to induce LTD. The conjunctive stimulation protocol is composed of 300 single PF stimuli in conjunction with depolarization pulses (− 80 to 0 mV, for 50 ms) repeated at 1 Hz. Series resistance and membrane resistance were monitored throughout the experiments, and the data were discarded if either of these resistances varied by more than 10%. The data were also discarded if the slope of the PF-EPSC amplitude averaged every minute during the initial recording for 10 min was larger than 2% or if the amplitude did not stabilize within 20 min after the onset of the whole-cell conditions^34,36,59^. The PF-EPSC amplitude was normalized by the mean value observed for 10 min before the CJS.

### Ca^2+^ imaging

For intracellular Ca^2+^ imaging in PCs, a calcium-sensitive dye, Oregon Green BAPTA-1 (100 μM), was introduced into the cells through the patch pipette, and the concentration of EGTA in the pipette solution was decreased to 0.5 mM. Five to nine sequential confocal images (excitation at 488 nm) obtained at 3–4 µm z-axis intervals were acquired every 0.8 s using an upright microscope (BX51WI; OLYMPUS) equipped with a confocal scanning unit and an argon laser (FV300, OLYMPUS), and were projected onto a plane to obtain images of dendrites at 10-s intervals. Nitric-oxide (NO) induced Ca^2+^ release (NICR) was induced by bath application of an NO donor, NOC7 (300 μM), as previously described^36^. Values are normalized with F0, the average value before drug application.

### ER staining

The morphology and distribution of the rough endoplasmic reticulum (ER) were analyzed by ER staining. Cerebellar slices were incubated with 1 μM ER-tracker red (Thermo Fisher Scientific) dissolved in the standard bathing solution for 30 min. Five to nine sequential confocal images were obtained and projected onto a plane to obtain images of PCs.

### Real-time PCR

Relative contents of type 1 inositol 1,4,5-tris phosphate receptor (IP3R1), type 1 ryanodine receptor (RyR1) and type 2 sarco/endoplasmic reticulum Ca^2+^-ATPase (SERCA2) were quantified by real-time PCR, according to the protocol in our previous study^37^. Total RNA preparations from 5 mice in each genotype were used as templates for cDNA synthesis (ReverTra ACE qPCR-RT kit, TOYOBO, Japan). The mRNA content was analyzed using a real-time PCR system according to the manufacturer’s instructions (Thermal Cycler TP800, TaKaRa Bio, Japan). The cycle threshold (Ct) was determined from the cDNA amplification curve as an index for relative mRNA content in each reaction.

### Western blot

Mouse brains were homogenized in a lysis buffer: 50 mM Tris–HCl (pH 8.0), 150 mM NaCl, 10 mM NaF, 1% Triton X-100, 0.5% sodium deoxycholate, 0.1% SDS and protease inhibitor mixture (Complete; Roche, Mannheim, Germany) to obtain cerebellar lysate. In some experiments, the cerebellum was homogenized in homogenize buffer [0.32 M sucrose, 1 mM EDTA, 1 mM EGTA, and 10 mM Tris (pH 7.4)]. The homogenate was centrifuged at 1000×*g* for 10 min, and the supernatant was further centrifuged at 10,000×*g* for 15 min. The supernatant was centrifuged again at 165,000×*g* for 120 min, and we used the pellet as cerebellar microsomal fraction. For western blot analysis, the total protein in the cerebellar lysate or microsomal fraction was separated by SDS-PAGE and blotted onto a polyvinylidene difluoride (PVDF) membrane. The membranes were incubated with primary antibodies (anti-RyR1 and anti-ShcB, whose specificity were confirmed in our previous studies^17,34^; anti-IP3R-1 (PA3-901, Affinity Bioreagents, Golden, CO, USA.); anti-SERCA2 (sc8905, Santa Cruz Biotechnology), anti-neuronal nitric oxide synthase (nNOS; ab1376, Abcam PLC, Cambridge, UK); anti-immunoglobulin heavy chain-binding protein (BiP; #610979, BD Biosciences, Franklin Lakes, NJ, USA) and anti-β-actin (#4967, Cell Signaling Technology, Danvers, MA, USA)) and proteins were detected by HRP-conjugated secondary antibodies using the ECL detection kit (GE Healthcare, Chicago, IL, USA). Because molecular weights of ShcB and BiP signals are close, the membranes were incubated with anti-BiP after deprobe of anti-ShcB for 40 min at 50 °C in the solution containing 2% SDS, 62.5 mM Tris–HCl (pH 8.0) and 100 mM 2-mercaptoethanol.

### Immunoprecipitation analysis

Proteins in cerebellar lysate, prepared as described above, were precipitated with acetone at -20 °C, and the pellet was resuspended in lysis buffer containing 25 mM Tris–HCl (pH 7.5), 100 mM NaCl, 2 μM EDTA, 0.05% (v/v) TritonX-100, protease inhibitor cocktails and calpain inhibitor I. For coimmunoprecipitations, 2 μl of anti-SERCA2 polyclonal antibody (sc-8905, Santa Cruz Biotechnology) was added to the lysate and allowed to bind to the antibody by shaking at 4 °C overnight, before addition of 40 μl preequilibrated Protein A/G sepharose (abcam). After shaking at 4 °C for another 1 h, beads were washed 3 times in lysis buffer and bound protein was eluted at 95 °C for 5 min in 50 μl of SDS-loading buffer. The signal for ShcB protein was detected by western blot analysis, as described above.

### SERCA activity assay

SERCA activity was analyzed using the EnzChek Phosphate Assay Kit (Thermo Fisher Scientific, MA, U.S.A.). Briefly, the homogenates from the cerebellum and kidney were incubated with ATP for 60 min at 36 °C with or without thapsigargin, an SERCA inhibitor. Then, the concentration of the enzymatic product by ATPase, inorganic phosphate, was analyzed by the concentration of the ribose 1-phosphate and 2-amino-6-mercapto-7-methylpurine product converted from the 2-amino-6-mercapto-7-methylpurine riboside (MESG) substrate by the purine nucleoside phosphorylase (PNP) in the presence of inorganic phosphate. SERCA activity was determined by subtraction of inorganic phosphate production with thapsigargin from that without thapsigargin.

### Preparation and infection of lentiviral vectors

Mouse ShcB cDNA^14^ was cloned into CSII-CMV-MCS-IRES2-mRFP1 to generate the transgene encoding ShcB-IRES-mRFP1. The vector plasmid carrying the transgene for ShcB-IRES-mRFP1 or mRFP1 in addition to packaging plasmids, cCAG-HIVgp and pCMV-VSV-G-RSV-Rev, was cotransfected into 293 T cells to prepare the lentiviral vectors. After the incubation for 48 h at 37℃, the medium was centrifuged at 50,000 g for 2 h to precipitate virus particles. The lentiviral titer was approximately 1 × 10^9^/ ml. For in vivo infection with viral particles, ShcB-KO mice (4-weeks old) were anesthetized with pentobarbital, and through an incision in the scalp, a small piece of the occipital bone and the dura covering the surface of cerebellar lobule VII were removed. Then, solutions containing viral particles carrying the transgenes were injected into lobule VII of the cerebellar cortex with a microglass needle (tip diameter of 20–40 μm) attached to a manipulator^34,59^. A volume of approximately 1 μl was delivered unilaterally within 5–10 min by air pressure. After the injection, the scalp was sutured. Approximately 8 weeks after the injection, Ca^2+^ release was analyzed from PCs in a virus-injected area of ShcB-KO cerebellar slices. The vector plasmids (CSII-CMV-MCS-IRES2-mRFP1 and CSII-CMV-mRFP1), packaging plasmids (cCAG-HIVgp and pCMV-VSV-G-RSV-Rev) and 293 T cells were provided from RIKEN BRC through the National Bio-Resource Project of the MEXT, Japan.

### Statistical analysis

We tested statistical significance using Student’s t-test or one-way ANOVA, followed by Dunnett’s test. Values are presented as mean ± SEM unless otherwise stated. Differences were considered significant when *p* < 0.05.

## Supplementary information

Supplementary file1

## References

[1] Berridge MJ (2002). The endoplasmic reticulum: a multifunctional signaling organelle. Cell Calcium.

[2] Berridge MJ, Lipp P, Bootman MD (2000). The versatility and universality of calcium signalling. Nat. Rev. Mol. Cell Biol..

[3] Clapham DE (2007). Calcium signaling. Cell.

[4] Mattson MP (2000). Calcium signaling in the ER: its role in neuronal plasticity and neurodegenerative disorders. Trends Neurosci..

[5] Paschen W, Mengesdorf T (2005). Endoplasmic reticulum stress response and neurodegeneration. Cell Calcium.

[6] Verkhratsky A (2005). Physiology and pathophysiology of the calcium store in the endoplasmic reticulum of neurons. Physiol. Rev..

[7] Xu CY, Bailly-Maitre B, Reed JC (2005). Endoplasmic reticulum stress: cell life and death decisions. J. Clin. Investig..

[8] Verkhratsky AJ, Petersen OH (1998). Neuronal calcium stores. Cell Calcium.

[9] Pawson T (2007). Dynamic control of signaling by modular adaptor proteins. Curr. Opin. Cell Biol..

[10] Algenstaedt PM, Antonetti DA, Yaffe MB, Kahn CR (1997). Insulin receptor substrate proteins create a link between the tyrosine phosphorylation cascade and the Ca^2+^-ATPases in muscle and heart. J. Biol. Chem..

[11] Lencesova L, O'Neill A, Resneck WG, Bloch RJ, Blaustein MP (2004). Plasma membrane-cytoskeleton-endoplasmic reticulum complexes in neurons and astrocytes. J. Biol. Chem..

[12] Manning G, Young SL, Miller WT, Zhai Y (2008). The protist, Monosiga brevicollis, has a tyrosine kinase signaling network more elaborate and diverse than found in any known metazoan. Proc. Natl. Acad. Sci. U.S.A..

[13] Wills MKB, Jones N (2012). Teaching an old dogma new tricks: twenty years of Shc adaptor signalling. Biochem. J..

[14] Kojima T (2001). Genomic organization of the Shc-related phosphotyrosine adapters and characterization of the full-length Sck/ShcB: Specific association of p68-Sck/ShcB with pp135. Biochem. Biophys. Res. Commun..

[15] Conti L (2001). Shc signaling in differentiating neural progenitor cells. Nat. Neurosci..

[16] Miyamoto Y (2005). Hippocampal synaptic modulation by the phosphotyrosine adapter protein ShcC/N-Shc via interaction with the NMDA receptor. J. Neurosci..

[17] Sakai R (2000). The mammalian ShcB and ShcC phosphotyrosine docking proteins function in the maturation of sensory and sympathetic neurons. Neuron.

[18] Conti L (1997). Expression and activation of SH2/PTB-containing ShcA adaptor protein reflects the pattern of neurogenesis in the mammalian brain. Proc. Natl. Acad. Sci. U.S.A..

[19] Nakamura T, Muraoka S, Sanokawa R, Mori N (1998). N-Shc and Sck, two neuronally expressed Shc adapter homologs—their differential regional expression in the brain and roles in neurotrophin and Src signaling. J. Biol. Chem..

[20] O'Bryan JP, Zhou SY, Cantley L, Der CJ, Pawson T (1996). A mammalian adaptor protein with conserved Src homology 2 and phosphotyrosine-binding domains is related to Shc and is specifically expressed in the brain. Proc. Natl. Acad. Sci. U.S.A..

[21] Nakamura T (1996). N-Shc: a neural-specific adapter molecule that mediates signaling from neurotrophin/Trk to Ras/MAPK pathway. Oncogene.

[22] Robeson HN (2019). Localization of phosphotyrosine adaptor protein ShcD/SHC4 in the adult rat central nervous system. BMC Neurosci..

[23] Lai KMV, Pawson T (2000). The ShcA phosphotyrosine docking protein sensitizes cardiovascular signaling in the mouse embryo. Genes Dev..

[24] Cattaneo E, Pelicci PG (1998). Emerging roles for SH2/PTB-containing Shc adaptor proteins in the developing mammalian brain. Trends Neurosci..

[25] Ravichandran KS (2001). Signaling via Shc family adapter proteins. Oncogene.

[26] Nakamura T (2002). Discrimination between phosphotyrosine-mediated signaling properties of conventional and neuronal Shc adapter molecules. Oncogene.

[27] Ponti G (2005). Comparative expression profiles of ShcB and ShcC phosphotyrosine adapter molecules in the adult brain. Neuroscience.

[28] McCormick DA, Thompson RF (1984). Cerebellum - Essential involvement in the classically-conditioned eyelid response. Science.

[29] Ito M (2002). The molecular organization of cerebellar long-term depression. Nat. Rev. Neurosci..

[30] Linden DJ, Connor JA (1995). Long-term synaptic depression. Annu. Rev. Neurosci..

[31] Maejima T, Hashimoto K, Yoshida T, Aiba A, Kano M (2001). Presynaptic inhibition caused by retrograde signal from metabotropic glutamate to cannabinoid receptors. Neuron.

[32] Bliss TV, Collingridge GL (1993). A synaptic model of memory: long-term potentiation in the hippocampus. Nature.

[33] Finch EA, Augustine GJ (1998). Local calcium signalling by inositol-1,4, 5-trisphosphate in Purkinje cell dendrites. Nature.

[34] Kakizawa S (2007). Junctophilin-mediated channel crosstalk essential for cerebellar synaptic plasticity. EMBO J..

[35] Kakizawa S (2013). Nitric oxide-induced calcium release: activation of type 1 ryanodine receptor, a calcium release channel, through non-enzymatic post-translational modification by nitric oxide. Front. Endocrinol..

[36] Kakizawa S (2012). Nitric oxide-induced calcium release via ryanodine receptors regulates neuronal function. EMBO J..

[37] Mikami Y (2016). Nitric oxide-induced activation of the type 1 ryanodine receptor is critical for epileptic seizure-induced neuronal cell death. EBioMedicine.

[38] Mori F, Fukaya M, Abe H, Wakabayashi K, Watanabe M (2000). Developmental changes in expression of the three ryanodine receptor mRNAs in the mouse brain. Neurosci. Lett..

[39] Wojcikiewicz RJH (1995). Tipe-I, type-II, and type-III inositol 1,4,5-trisphosphate receptors are unequally susceptible to down-regulation and are expressed in markedly different proportions in different cell-types. J. Biol. Chem..

[40] Usachev YM, Marsh AJ, Johanns TM, Lemke MM, Thayer SA (2006). Activation of protein kinase C in sensory neurons accelerates Ca^2+^ uptake into the endoplasmic reticulum. J. Neurosci..

[41] Brini M, Carafoli E (2009). Calcium pumps in health and disease. Physiol. Rev..

[42] Mata AM, Sepulveda MR (2005). Calcium pumps in the central nervous system. Brain Res. Rev..

[43] Bhupathy P, Babu GJ, Periasamy M (2007). Sarcolipin and phospholamban as regulators of cardiac sarcoplasmic reticulum Ca^2+^ ATPase. J. Mol. Cell. Cardiol..

[44] Stammers AN (2015). The regulation of sarco(endo)plasmic reticulum calcium-ATPases (SERCA). Can. J. Physiol. Pharmacol..

[45] MacLennan DH, Kranias EG (2003). Phospholamban: a crucial regulator of cardiac contractility. Nat. Rev. Mol. Cell Biol..

[46] Asahi M (2003). Sarcolipin regulates sarco(endo)plasmic reticulum Ca^2+^-ATPase (SERCA) by binding to transmembrane helices alone or in association with phospholamban. Proc. Natl. Acad. Sci. U.S.A..

[47] Vangheluwe P, Raeymaekers L, Dode L, Wuytack F (2005). Modulating sarco(endo)plasmic reticulum Ca(2+) ATPase 2 (SERCA2) activity: cell biological implications. Cell Calcium.

[48] Christian KM, Thompson RF (2003). Neural substrates of eyeblink conditioning: acquisition and retention. Learn. Mem..

[49] Ito M (2001). Cerebellar long-term depression: characterization, signal transduction, and functional roles. Physiol. Rev..

[50] Kakizawa S (2005). Maintenance of presynaptic function by AMPA receptor-mediated excitatory postsynaptic activity in adult brain. Proc. Natl. Acad. Sci. U.S.A..

[51] Kakizawa S, Yamasaki M, Watanabe M, Kano M (2000). Critical period for activity-dependent synapse elimination in developing cerebellum. J. Neurosci..

[52] Kishimoto Y (2002). mGluR1 in cerebellar Purkinje cells is required for normal association of temporally contiguous stimuli in classical conditioning. Eur. J. Neurosci..

[53] Kishimoto Y (2013). Age-dependent impairment of eyeblink conditioning in prion protein-deficient mice. PLoS ONE.

[54] Anderson KD, Abdul M, Steward O (2004). Quantitative assessment of deficits and recovery of forelimb motor function after cervical spinal cord injury in mice. Exp. Neurol..

[55] Susick LL (2014). Adenylyl cylases 1 and 8 mediate select striatal-dependent behaviors and sensitivity to ethanol stimulation in the adolescent period following acute neonatal ethanol exposure. Behav. Brain Res..

[56] Miyamoto Y (2002). Lower sensitivity to stress and altered monoaminergic neuronal function in mice lacking the NMDA receptor epsilon 4 subunit. J. Neurosci..

[57] Noda Y, Yamada K, Furukawa H, Nabeshima T (1995). Enhancement of immobility in a forced swimming test by subacute or repeated treatment with phencyclidine: a new model of schizophrenia. Br. J. Pharmacol..

[58] Edwards FA, Konnerth A, Sakmann B, Takahashi T (1989). A thin slice preparation for patch clamps recordings from neurons of the mammalian central nervous-system. Pflugers Arch..

[59] Namiki S, Kakizawa S, Hirose K, Iino M (2005). NO signalling decodes frequency of neuronal activity and generates synapse-specific plasticity in mouse cerebellum. J. Physiol. (Lond.).

