# Supplementary material for: Functional maintenance of calcium store by ShcB adaptor protein in cerebellar Purkinje cells
Source: Sci Rep. 2020 Sep 2;10:14475. doi: 10.1038/s41598-020-71414-y (PMC7468156; doi:10.1038/s41598-020-71414-y)
Supplement: Supplementary file 1 — Supplementary file1 [file 41598_2020_71414_MOESM1_ESM.docx]

**Supplementary Information**

**Functional maintenance of calcium store by ShcB adaptor protein in cerebellar Purkinje cells**

**Sho Kakizawa, Yasushi Kishimoto, Shinichiro Yamamoto, Kazuko Onga, Kunihiko Yasuda, Yoshiaki Miyamoto, Masahiko Watanabe, Ryuichi Sakai and Nozomu Mori**





**Supplementary Figure S1. Full length images of western blotting.**

Protein expression of ShcB and actin (A; related to Fig. 1B), RyR1 and BiP (B; related to Fig. 5B), IP3R and BiP (C; related to Fig. 5B) and SERCA2, BiP and actin (D, related to Fig 7D). Western-blot signals (E, related to Fig 7F) detected by anti-SERCA2 antiserum, anti-ShcB antiserum or anti-actin antiserum after IP by mouse anti-SERCA2 antiserum (IP: SERCA2) or by normal mouse IgG (IP: normal IgG). Lysates from WT and ShcB-KO cerebellum without IP (Input) were also applied. RyR1: type 1 ryanodine receptor, IP_3_R1: type 1 inositol-1,4,5-phosphate receptor, Bip: immunoglobulin heavy chain-binding protein, SERCA2: sarco/endoplasmic reticular Ca^2+^-ATPase, IP: immunoprecipitation.
